# 3D printable hyaluronic acid-based hydrogel for its potential application as a bioink in tissue engineering

**DOI:** 10.1186/s40824-018-0152-8

**Published:** 2019-02-06

**Authors:** Insup Noh, Nahye Kim, Hao Nguyen Tran, Jaehoo Lee, Chibum Lee

**Affiliations:** 1 Department of Chemical and Biomolecular Engineering, Seoul National University of Science and Technology, Seoul, 01811 Republic of Korea; 2 Convergence Institute of Biomedical Engineering and Biomaterials, Seoul National University of Science and Technology, Seoul, 01811 Republic of Korea; 3 Department of Mechanical System Design Engineering, Seoul National University of Science and Technology, Seoul, 01811 Republic of Korea

**Keywords:** 3D bioprinting, Hyaluronic acid, Gelatin, Biocompatible, Tissue engineering

## Abstract

**Background:**

After recognition of 3D printing and injectable hydrogel as a critical issue in tissue/organ engineering and regenerative medicine society, many hydrogels as bioinks have been developed worldwide by using polymeric biomaterials such as gelatin, alginate, hyaluronic acid and others. Even though some gels have shown good performances in 3D bioprinting, still their performances do not meet the requirements enough to be used as a bioink in tissue engineering.

**Method:**

In this study, a hydrogel consisting of three biocompatible biomaterials such as hyaluronic acid (HA), hydroxyethyl acrylate (HEA) and gelatin-methacryloyl, i.e. HA-g-pHEA-gelatin gel, has been evaluated for its possibility as a bioprinting gel, a bioink. Hydrogel synthesis was obtained by graft polymerization of HEA to HA and then grafting of gelatin- methacryloyl via radical polymerization mechanism. Physical and biological properties of the HA-based hydrogels fabricated with different concentrations of methacrylic anhydride (6 and 8%) for gelatin-methacryloylation have been evaluated such as swelling, rheology, morphology, cell compatibility, and delivery of small molecular dimethyloxalylglycine. Printings of HA-g-pHEA-Gelatin gel and its bioink with bone cell loaded in lattice forms were also evaluated by using home-built multi-material (3D bio-) printing system.

**Conclusion:**

The experimental results demonstrated that the HA-g-pHEA-gelatin hydrogel showed both stable rheology properties and excellent biocompatibility, and the gel showed printability in good shape. The bone cells in bioinks of the lattice-printed scaffolds were viable. This study showed HA-g-pHEA-Gelatin gel’s potential as a bioink or its tissue engineering applications in injectable and 3D bioprinting forms.

## Background

3D Bioprinting has been recognized as one of the latest biotechnologies, which is highly used in tissue engineering and regenerative medicine to develop complex artificial tissue and organ structures to mimic native organs and tissues [1–7]. The bioprinting involves additive deposition of cells-loaded hydrogels in a predetermined structural architecture to regenerate functional and site-specific tissues or organs [4, 8]. This technique integrates hydrogels, live cells and controlled printing systems to create complex morphological structures, and has demonstrated precise control of the targeted structures than any currently available other methods [9–11]. Hence, very complex structures with controlled porosity, permeability and mechanical properties similar to patient’s own tissues and organs are possible by bioprinting [1, 2], with computer-aided design (CAD) and complex geometrical data such as magnetic resonance imaging (MRI), X-ray imaging and micro-computerized tomography scan (μ-CT-scan) [1]. Even though there are many advantages of 3D bioprinting in biomedical field such as personalized patient-specific designs, high precision, on-demand creation of complex structures within a short time and with low cost, incorporation of cells in the printed scaffolds and hydrogels should be possible for tissue regeneration [5, 6].

Bioinks have recently attracted high interesting for development of functional tissues and organs in 3D bioprinting tissue engineering. Among bioink biomaterials, gelatin-methacrylates, agarose, alginate, collagen, chitin, silk, hyaluronic acid, cellulose and their mixtures have been employed as important bioink materials by using diverse cross-linking mechanisms such as click chemistry, ionic/hydrogen bonding in alginate bioinks and chemical bonding in alginate-methacrylate bioinks via radical initiators [12–21]. Alginate bioinks showed better cell encapsulation and survival, but their post-printing morphological stability is a critical issue to be resolved. Gelatin-methacrylate and modified collagen have been quickly obtained as bioinks by using cross-linking agents such as glutaldehyde, 1-ethyl-3-(3-dimethylaminopropyl) carbodiimide (EDC), Eosin-Y and igarcure and etc. [22–25]. Even though morphological stability is excellent in this method, still removal of cross-linking agents and cytotoxicity should be solved in its final applications to patients. Furthermore, when applying the new bioink as in micro-extrusion printing, there are many huddles and challenges that needed to be overcome. Hyaluronic acid (HA) is progressively being applied for biomedical applications for decades since it is naturally biocompatible and indispensable in regulating cellular behaviors [24, 26, 27]. Again, though tissue functions of HA gel including cell migration, angiogenesis, viability and proliferation, its post-printing shape stability is weak, thus making its applications in bioprinting as bioinks.

The major challenges of bioinks are encapsulation of cells, bioprintability, biocompability, minimal cytotoxicity and high post-printing morphological stability, which maintains its shapes under wet condition to support cell adhesion and proliferation by modifying their chemical structures [28–30]. Herein, the purpose of our study was to evaluate the physical and biological properties of our newly developed hydrogel, as well as cell encapsulation in the gel. We recently reported a terpolymeric HA-HEA-PEGDA hydrogel to improve hydrogel’s mechanical stability by employing biocompatible polymers [26]. To increase its cellular interaction with cells we adopted gelatin as a component of terpolymer gel, synthesizing a HA-HEA-gelatin hydrogel. Gelatin methacryloyl is an attractive photo-crsslinking polymer which is synthesized from chemical modification of gelatin with methacrylic anhydride. This terpolymeric HA-HEA-Gelatin hydrogel has been reported as our new work (31). In this study after evaluation of its diverse physical and biological properties such as swelling, drug release and rheological properties, we tested its printing and bioink printing ability to evaluate its potential possibility as bioinks by using home-built multi-material (3D bio-) printing system. The cells in bioprinted lattice scaffold were viable and the post-printed morphology was stable, indicating a possibility of its usage as a bioink.

## Methods

### Materials

Sodium salt of hyaluronic acid (HA, M.W. = 1660 kDa, PDI = 3.974) was graciously donated from Hanmi Pharm. Co. Ltd., Korea, and then chemically modified for gel synthesis [26, 30]. Potassium peroxodisulfate (KPS), gelatin (source: bovine skin), 2-hydroxyethyl acrylate (HEA), methacrylic anhydride (MA), dimethyloxaloylglycine (DMOG) and Dulbecco’s Modified Eagle Medium (DMEM) were purchased from Sigma Aldrich Chemical Co. (St. Luis, MO, USA, Germany and China). Tissue culture agents such as fetal bovine serum (FBS, Biotechnics Research, Mission Viejo, CA, USA), penicillin-streptomycin (Lonza, Seoul, Korea), 0.05% trypsin-EDTA-1X (Gibco-Life Technologies, Carlsbad, California, USA), and live & dead viability/cytotoxicity kit for mammalian cells (Invitrogen, Carlsbad, CA, USA) were purchased and used. Osteoblast precursor cell line derived from *Mus musculus* (mouse) calvaria, P9, was used for biocompatibility tests and distilled water (DW) was employed for all experiments.

### Synthesis

#### Synthesis of gelatin-methacryloyl (gelatin-MA)

Synthesis of gelatin-methacrylation was performed by slight modification of the protocol described in the literature [29, 31]. At first, gelatin (1 g) was dissolved in 50 mL of phosphate buffer (pH 7.5) at 50 °C, and then methacrylic anhydride was added dropwise and stirred at 400 rpm. Different concentrations of methacrylic anhydride such as 4, 6 and 8% were employed to control its viscosity for printing. After 3 h, the reaction mixture was diluted with 50 mL of phosphate buffer solution (pH 7.5) and dialyzed for 4 days against distilled water at 40 °C for purification. The reaction product was freeze-dried and termed as Gelatin-MA in this study. The degree of substitution (DS) is determined by the method described in the literature and reported in our previous report [31].

#### Preparation of HA-based hydrogel

HA-based hydrogel was synthesized as below. Firstly, a homogeneous solution of HA (0.25 g, 0.623 × 10^− 3^ mol with respect to the molecular weight of one repeating unit) was added in 60 mL of distilled water into a 2-neck round bottom flask at room temperature. Next, the HA solution was located in a digital glass oil bath (LK Lab Korea, Korea) at 75 °C and stirred with a stirrer at 400 rpm. After 2 h, nitrogen gas was pursed into the solution for 30 min to make an inert atmosphere. After that, 5 mL aqueous KPS solution (0.0025 g, 0.0092 × 10^− 3^ mol) as initiator was mixed to the HA solution. After 20 min, 3 ml of HEA (17.41 × 10^− 3^ mol) as a monomer was poured to the mixture. When the viscosity of the solution changed, 5 mL aqueous Gelatin-MA solution (0.25 g) as a crosslinker was added and the reaction was processed for another 3 h, thus obtaining a gel-like product. Then, the gel-like product was purified by dialysis in distilled water at 25 °C for 2 days. The purified product (HA-g-pHEA-x-Gelatin-MA) was dried at lyophilizer at − 56 °C for 7 days, and used for characterizations and applications.

### Morphological characterizations of hydrogel by digital and scanning electron microscope

After observation of hydrogel’s morphological images with digital camera, their morphological images of bioprinted hydrogel and bioinks, their images were taken by light microscopy (Olympus, Japan). The morphological images of hydrogels were also observed with SEM at different magnifications under inert environment after drying in − 78 C lyophilizer and then platinum coating for 1 min. The dry gel samples were fixed in advance on double sided tape on aluminum.

### Swelling study

The % swelling of the dried HA-g-pHEA-Gelatin gel was measured gravimetrically. In brief, 0.5 mL of lyophilized HA-g-pHEA-Gelatin gel sample was immersed in 20 mL buffer solution at pH 7.4 at 37 °C for 14 h. After a regular interval (1 h), the water-soaked sample was taken out from solution, surface water was blotted off by a tissue paper and reweighed until an equilibrium weight was reached. The % swelling was measured by employing the Eq. (1):

$$ \mathbf{Swelling}\ \left(\%\right)=\frac{\mathbf{Wt}.\mathbf{of}\ \mathbf{wet}\ \mathbf{sam}\mathbf{ple}-\mathbf{Wt}.\mathbf{of}\ \mathbf{dried}\ \mathbf{sample}}{\mathbf{Wt}.\mathbf{of}\ \mathbf{dried}\ \mathbf{sam}\mathrm{p}\mathbf{le}}\times \mathbf{100}\left(\%\right)\ \left(\mathbf{1}\right) $$

### 3D printing of HA-g-pHEA-gelatin hydrogels

Home-built multi-material (3D bio-) printing system (Seoul Tech) introduced in the previous paper [32] and equipped with rotating dual pressure-driven extruders and heating facilities was used to print the hydrogels. 3D gel structures with different templates and infills were designed using Solid works software (Dassault Systems SolidWorks Corp, USA), and the G-codes for the stereolithography (STL) files were generated using a slicing software (Simplify3D version 4.0, USA). The cross-linked HA-g-pHEA-Gelatin hydrogels (6 ml) were loaded inside the plastic syringe (10 ml, Musashi Engineering Inc., Korea) attached with a plastic orifice (25 gauge). The HA-g-pHEA-Gelatin gels in the syringe needle was placed proximal to the stage with the substrate by adjusting the Z-axis (syringe holder), X- and Y-axis (stage) using software. The pressure and temperature for printing were optimized by checking up the continuity and stability of the hydrogel extruded from the needle, and by varying other parameters such as printing pressure, temperature, nozzle and stage speed, and nozzle diameter. The optimized parameters were obtained as pressure 161 kPa, temperature 35 °C and speed 100 mm/min in our multi-material (3D bio) printing system. The HA-g-pHEA-Gelatin gels with and without MC3T3 cells were printed with 2 layers onto the glass coverslips (d = 1 cm). The live and dead images of the dispensed bone cells were acquired on day 3 after bioprinting, using a fluorescence microscope.

### In vitro bone cell study

#### Behaviours of MC3T3 bone cells in the HA-g-pHEA-gelatin hydrogel

HA-g-pHEA-Gelatin hydrogel was sterilized by autoclave at 121 °C for 15 min, then placed in 24-well plates (300 μL/well). MC3T3 bone cells with low passage were in vitro cultured in DMEM media containing 10% fetal bovine serum and 1% penicillin-streptomycin in an incubator at the conditions of 37 °C and 5% CO_2_ atmosphere until getting confluence. After that, MC3T3 bone cells were trypsinized and injected into HA-g-pHEA-Gelatin gel (1 mL) at a density of 1 × 10^5^ cells/wells. The bone cell-encapsulated hydrogels were cultured in 1 mL of DMEM (10% FBS and 1% penicillin-streptomycin). Culture medium was changed after 24 h of incubation and then after every 48 h. MC3T3 bone cells were also cultured on 24-well tissue culture plate (1 × 10^5^ cells/wells) and used as a control.

#### Live & Dead assay

Cell viability on HA-g-pHEA-Gelatin hydrogel was evaluated by the live and dead assay after in vitro bone cell culture for 7 days. Live and dead viability/cytotoxicity assay for bone cells was processed according to the protocol suggested by the vendor (Invitrogen, USA). 1 mL of cell suspension was obtained from the HA-g-pHEA-Gelatin hydrogel. Two times of PBS washing was employed, and then the assay solutions that was composed of 1.2 μL of 2 mM ethidium homodimer-1 and 0.3 μL of 4 mM calcein AM (dead and live stains, respectively) in 600 μL PBS. In vitro cell viability in gel was observed by a fluorescence microscope (Leica DMLB, Germany) after 30 min incubation in 37 °C in the 5% CO_2_ incubator.

### In vitro drug release study

#### Loading of DMOG in the HA-g-pHEA-gelatin gel

DMOG was loaded as a model small molecular weight bioactive molecule in the HA-g-pHEA-Gelatin gel. DMOG (0.00125 g, 0.0009 g, 0.00045 g per mL) were dissolved into 2 mL of distilled water in a Teflon vial. After that, 0.434 ± 0.0133 g of dried gel was immersed in the above DMOG solution and gently shaken using an orbital shaker (ROTAMAX 120, Heidolph, Germany) at room temperature for 48 h. Then, the loaded gel was taken out from the Teflon vial, rinsed with distilled water and dried in a lyophilizer at − 78 °C for 48 h. The amount of DMOGs in the supernatant solution was calculated by UV-Vis spectroscopy. Each test was performed in triplicate. The % DMOG loading efficiency was measured by the Eq. (2), (Das, Rameshbabu, et al., 2017).

$$ \mathrm{Loading}\ \mathrm{efficiency}\ \left(\%\right)=\frac{\mathrm{Wt}.\mathrm{of}\ \mathrm{DMOG}\ \mathrm{drug}\ \mathrm{in}\ \mathrm{gel}}{\mathrm{Wt}.\mathrm{of}\ \mathrm{dried}\ \mathrm{gel}\ \mathrm{taken}}\times 100\ \left(\%\right)\ (2) $$

#### In vitro DMOG release study

In vitro DMOG release studies from small molecular DMOG-loaded HA-g-pHEA-Gelatin gel were performed at pH 7.4, and 37 °C. Briefly, the small molecular DMOG loaded gels were put in the flasks containing 20 mL of buffer media (pH 7.4). After 1, 3, 6, 24, and 48 h, aliquots were taken out from flasks and absorbance was measured by a UV-Vis spectrophotometer (Model: BioMATE 3, Thermo Scientific, Madison, USA). After each measurement, old buffer solutions were replaced by new buffer solutions. The % DMOG release were calculated on the basis of standard DMOG solutions. Each test was performed in triplicate.

### Statistical analysis

All data were represented as mean ± standard deviation. Statistical significance was evaluated with one-way and multi-way ANOVA by using the SPSS 18.0 program (ver. 18.0, SPSS Inc., Chicago, IL, USA). The comparisons between two groups were performed by t-test, where, the significant level was *p* < 0.05.

## Results

### Synthesis of HA-g-pHEA-gelatin gel

The HA-g-pHEA-Gelatin gel was synthesized using HA as a biopolymer, HEA as a monomer, Gelatin-MA (0.25 g) as a crosslinker, and KPS as an initiator at 75 °C as reported in previous. [31]. In brief, sulphate anion radicals from KPS abstracted protons from hydroxyl groups of HA and then generated HA-macro-radicals. The reactive radicals of HA-g-pHEA reacts with methacrylate in Gelatin-MA. It was hypothesized that while all reactive sites were coupled with the one end of acrylamide site of Gelatin-MA, another site also connects another Gelatin-MA, thus acrylate group of Gelatin-MA took part in polymerization and formed a crosslinked network. The possible mechanism has been described in detail in a paper [31], by using the results of their chemical analyses such as 1H HR-MAS NMR, FTIR and TGA. We adopted this hydrogel with different concentrations of Gelatin-MA agents for the evaluations of both hydrogel and bioink its printability and in vitro cell viability in this study.

### Characterizations

#### Swelling

**Fig. 1:**
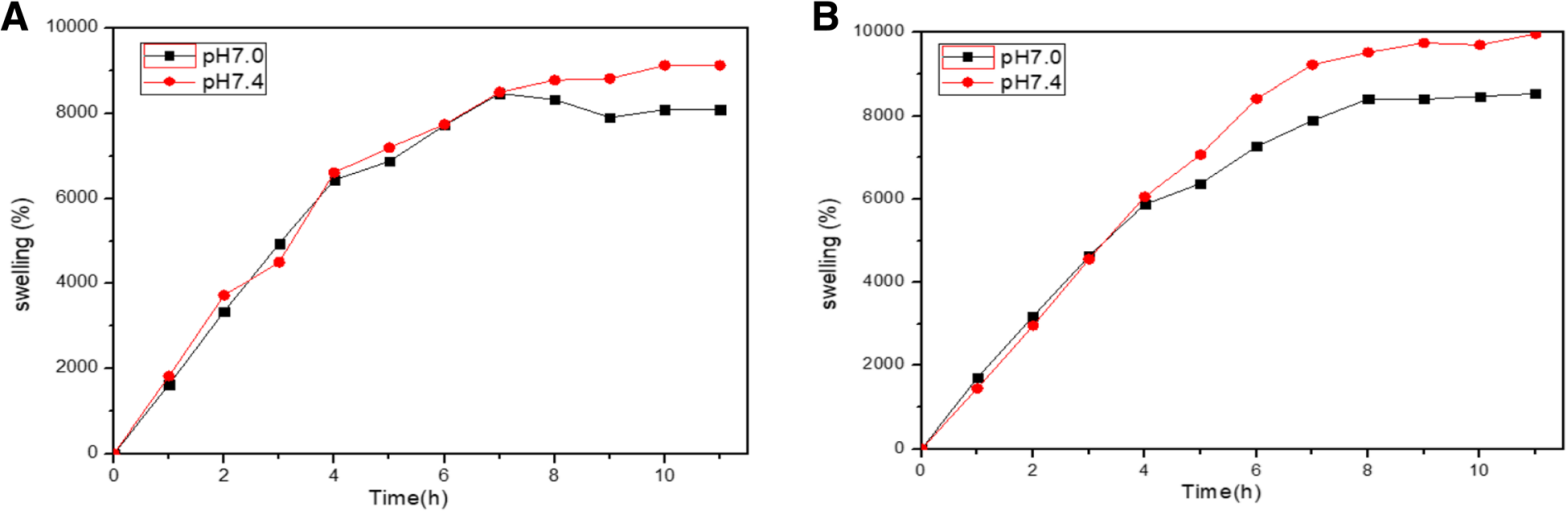
Swelling behaviors of HA-g-pHEA-Gelatin hydrogel at pH 7.0 and pH 7.4 and 37 °C, where Gelatin-MA were fabricated with 6% (**a**) and 8% (**b**) methacrylic anhydride, respectively

Figure 1 is the swelling test result of HA-g-pHEA-Gelatin gel (6 and 8% methacrylic anhydride) at pH 7.4 and 37 °C. The HA-g-pHEA-Gelatin gel attained its equilibrium state of swelling at about 8 h, which established full expansion of the hydrogel network. The swelling ability of the HA-g-pHEA-Gelatin gel is attributed to the presence of different hydrophilic functional groups (-COONa, -OH, -NH_2_, and -CONH-) in the terpolymeric network. The % swelling of the hydrogel (*w*/*v*) was approximately 80 to 100 times increase over dry weight when it reached an equilibrium in 10 h depending on both pHs and conditions of the synthesized hydrogels. pH 7.4 induced more swelling of hydrogel than pH 7.0 did from approximately 4 to 7 h after immersing in water.

#### Morphologies

**Fig. 2:**
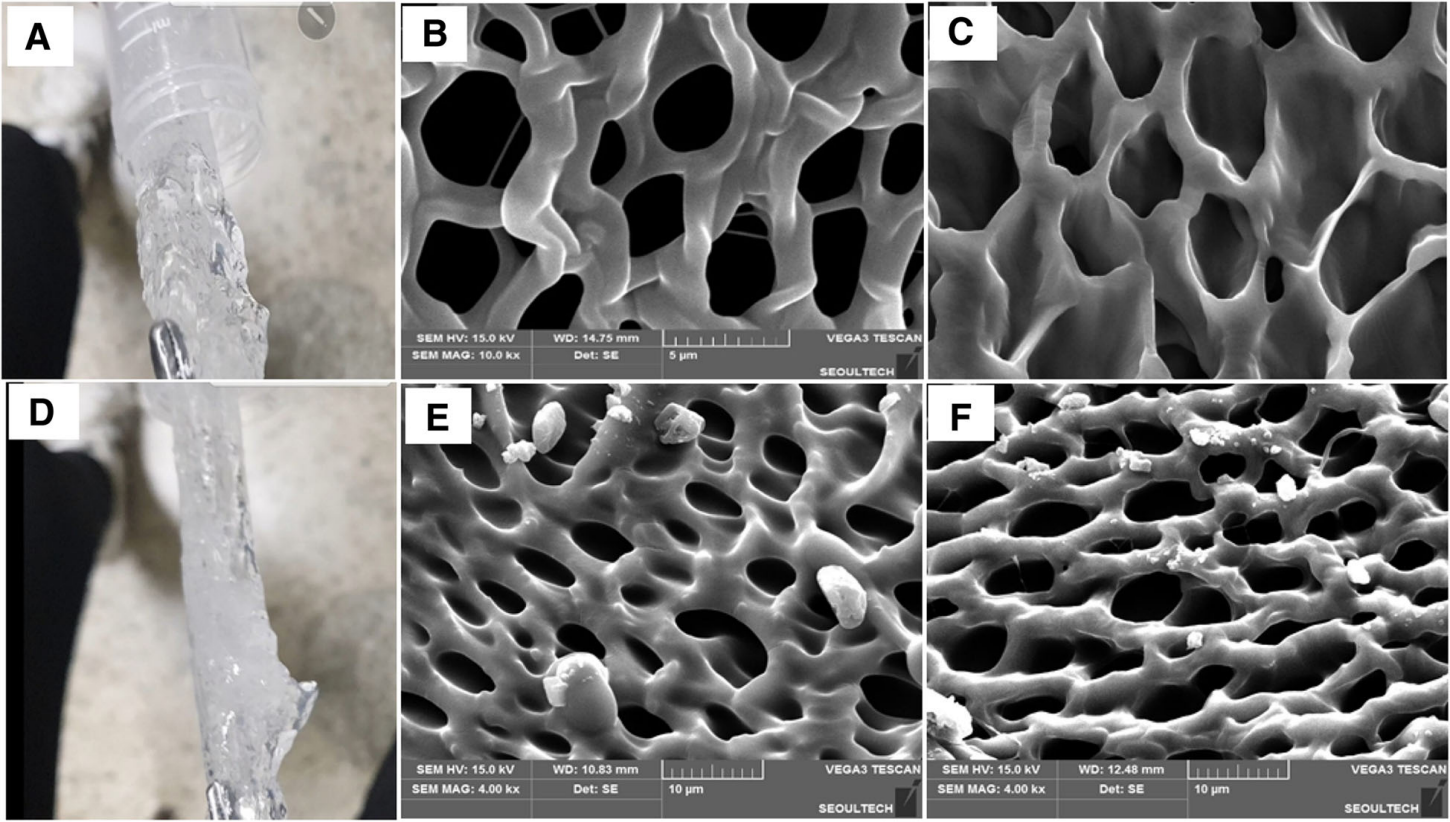
Digital (**a**, **d**) and surface (**b**, **e**) and cross-section (**c**, **f**) of scanning electron microscopy (**b**, **c**, **e** and **f**) morphologies of HA-g-pHEA-Gelatin hydrogel (**a**, **b** and **c**: 6% methacrylic anhydride and D, E and F: 8% methacrylic anhydride). The gel composition is 0.25 g HA, 3 mL HEA, 0.25 g Gelatin-MA, and the scale bars of (b and **c**) and (**e** and **f**) are 5 μm and 10 μm, respectively

From the digital images in Fig. 2, it is observed that the hydrogel synthesized with different Gelatin-MAs have different properties such as apparent shapes (Fig. 2-A and B), i.e. while the surface morphology of Gelatin-MA (6% methacrylic anhydride) showed more transparent shape (Fig. 2-A), that with Gelatin-MA (8% methacrylic anhydride) more opaque (Fig. 2-D). Even though there was difference in shapes, the morphology of both gels (Fig. 2-B, C) in SEM images showed similar pore sizes (3–5 μm in diameter) (Fig. 2-E, F), note different scale bars between the images of the 6 and 8% methacrylic anhydride employed gels.

#### Rheology

**Fig. 3:**
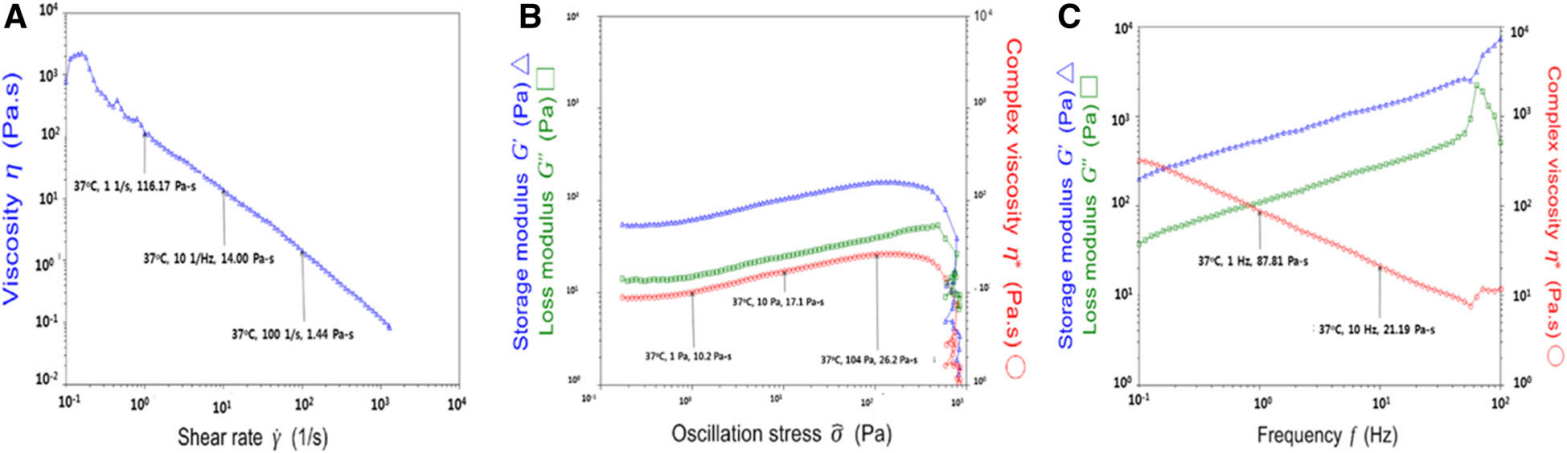
Rheological behaviors of HA-g-pHEA-Gelatin (6% methacrylic anhydride) hydrogel, where the relations of (**a**) viscosity change over shear rate; storage-loss modulus and complex viscosity over oscillation stress (**b**) and frequency (**c**)

Rheological properties of HA-g-pHEA-Gelatin (6% methacrylic anhydride) hydrogel were evaluated by measuring complex viscosity over shear rate (Fig. 3-A), as well as storage modulus and loss modulus over oscillation stress (Fig. 3-B) and frequency (Fig. 3-C), respectively. As shear rate increases from 0.1/s to 1000/s, its viscosity decreased from approximately 1100 to 0 Pa-sec. As oscillation stress increase to 400 Pa, complex viscosity, storage and loss modulus increased and disappeared. Their behaviors showed crossing of storage modulus and loss modulus at around 80 Hz, and its complex viscosity decreased accordingly.

#### Drug release

**Fig. 4:**
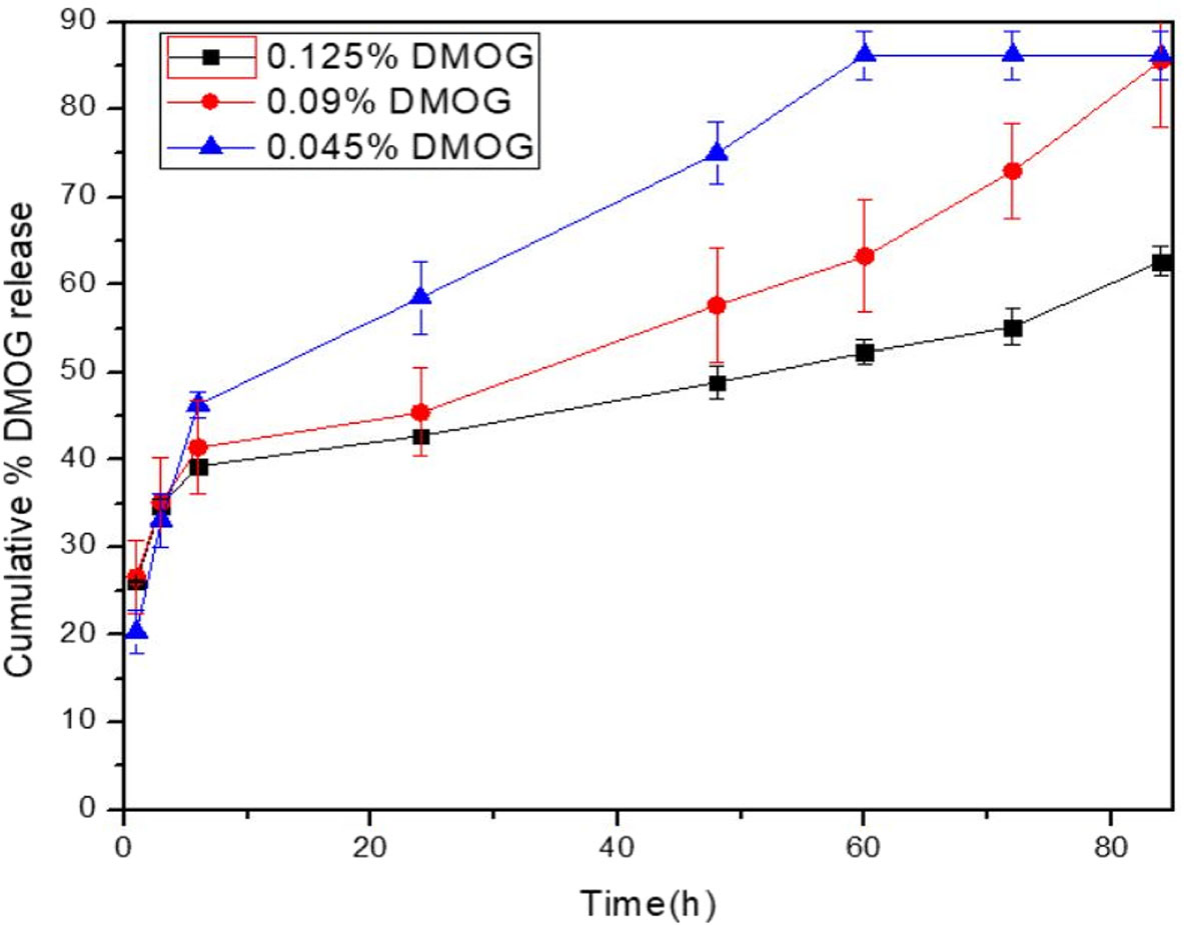
Release of DMOG drug in different amount from the HA-HEA-Gelatin hydrogel over time

Release of dimethyloxalylglycine (DMOG, MW 175) from the hydrogel was measured over time up to 180 h. MDOG is a cell-permeable prolyl-4-hydroxylase inhibitor, which upregulates hypoxia-inducible factor (HIF). We measured the behaviors of DMOG release from the hydrogel over time after loading 0.0025 g, 0.0018 g, 0.0009 g per 2 mL gel for 84 h. Initial bust release of DMOG was observed from the hydrogel, and its release lasted sustainably to 84 h in this study. Higher amount of DMOG loading induced longer time in its release, in specific 63, 86 and 86% for the 0.125, 0.09 and 0.045% DMOG-loaded gel (*w*/*v*), respectively (Fig. 4).

#### 3D printability test and bioink printing of the HA-HEA-gelatin hydrogel

**Fig. 5:**
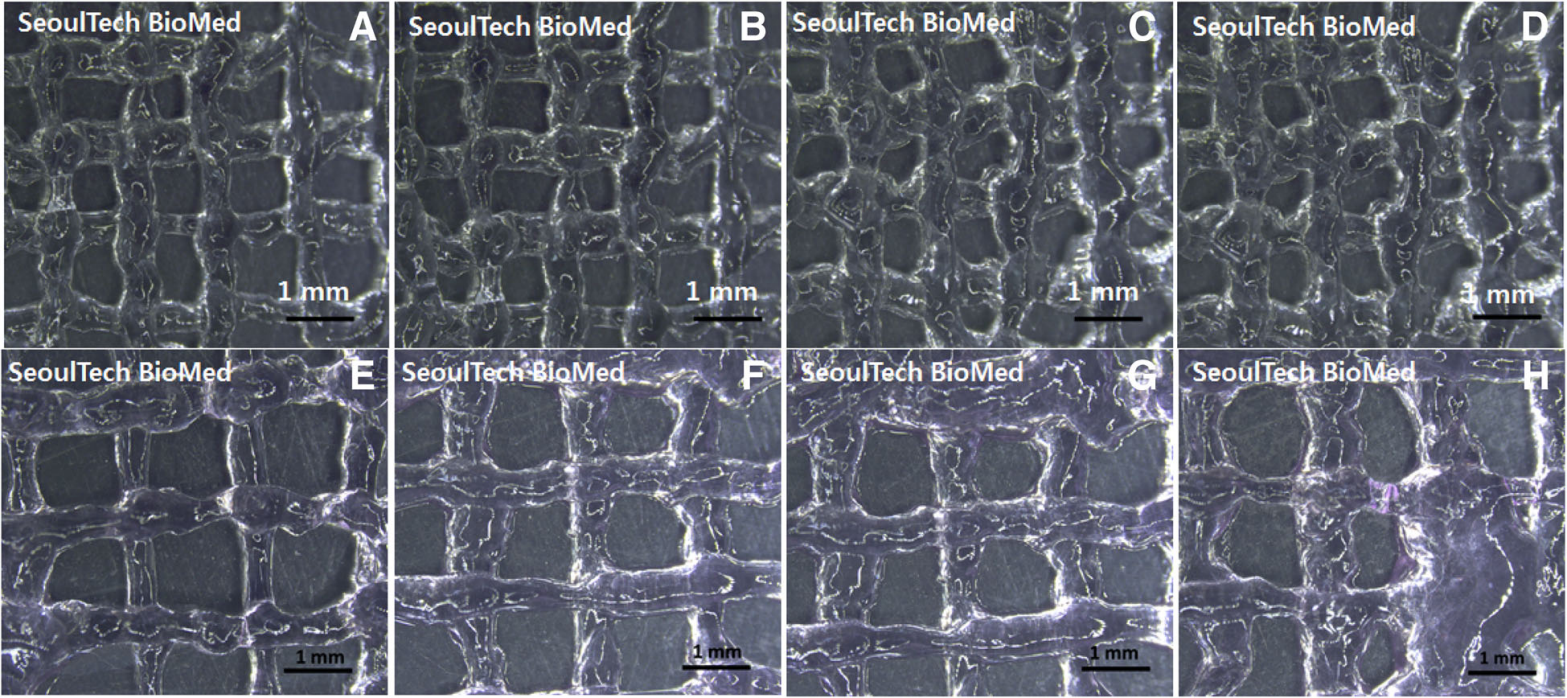
Optical images of printed HA-g-pHEA-Gelatin gel scaffolds with/without cells. (**a**, **b**, **c**, **d**) Non-cell loaded hydrogel and (**e**, **f**, **g**, **h**) cell-loaded bioinks were extruded from the nozzle by applying different air pressures of 450, 500, 550 and 600 kPa respectively

Printing of HA-g-pHEA-Gelatin gel with bone cells unloaded was performed in a lattice form by extruding it with different pressures of 450, 500, 550 and 600 kPa in our extrusion printing system (Fig. 5-a, b, c, d). While the printing lines were observed as approximately 500 to 700 μm, the distance between each line were measured as approximately 1 mm. Next, after incorporating bone cells in the hydrogel, i.e. forming a bioink, we printed out it in a lattice form at the same pressures of 450, 500, 550 and 600 kPa, respectively (Fig. 5-e, f, g, h). Increases in lines were observed after printing bioinks due to the loaded cells in the gel. Their printing lines increased to approximately 1 mm.

**Fig. 6:**
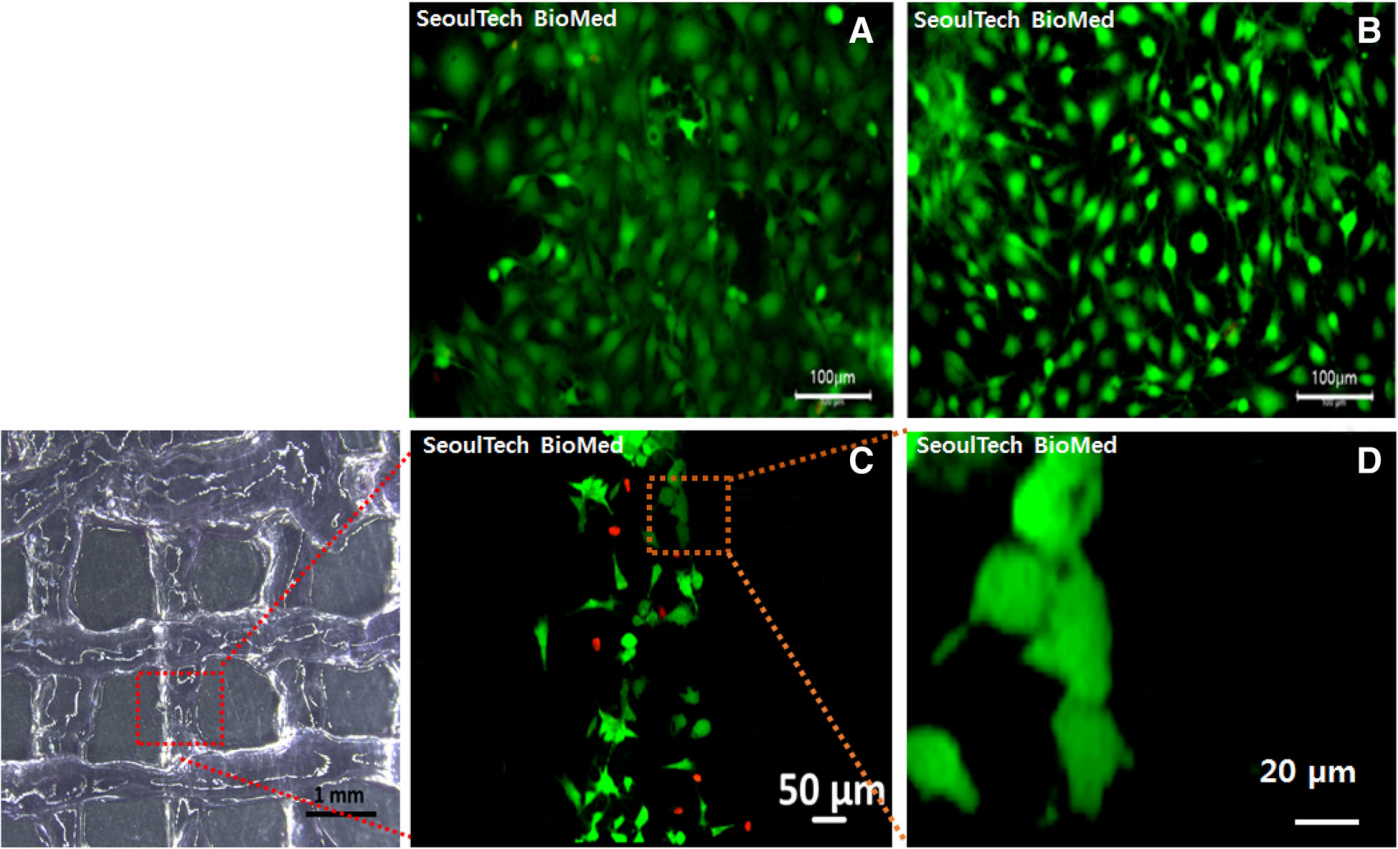
Live and dead assay results of the bone cells s in HA-g-pHEA-Gelatin (**a**, **b**) and 1 day in vitro cell culture after bioprinting of the HA-g-pHEA-Gelatin gel as a bioink (**c**, **d**)

Next we tested in vitro viability of the bone cells in both the hydrogel and bioinks after 3D printing (Fig. 6). Before printing, all the cells loaded in the hydrogel were viable and well proliferated with spreading (Fig. 6-A, B). After 3D printed, the results of bioink printing showed its printing lines with cells incorporated (Fig. 6-C and D) but small amount of cells died as shown in Fig. 6-C. The printed cells line of the bioink was observed approximately 500 μm in width.

## Discussion

Bioinks and injectable hydrogels are considered as a key issue in tissue/organ engineering and regenerative medicine society, and many bioink hydrogels have been developed worldwide by using biocompatible polymers such as gelatin, agarose, chitosan, alginate, hyaluronic acid, silk, fibrin and other natural polymers [3]. Biomaterial properties for bioinks include printability, mechanical/post-printing stability, controlled biodegradation, viscosity, modifiable functional side chain groups on the polymer. Biological requirements of bioinks include biocompatibility, which is not only non-toxic to the host tissues/cells, but also live cells’ viability inside bioink, cytocompatibility, and bioactivity of cells after bioprinting [3]. Diverse crosslinking methods have been also reported for bioprinting such as photochemical crosslinking, ionic bonding, hydrophobic interactions, hydrogen bonding, self-crosslinking such as Diels-Elders reaction, Michael type reaction [3, 33].

Some hydrogels have demonstrated good performances in their applications in 3D bioprinting, but their performances do not meet the bioink requirements in tissue engineering. Even though alginate gel has self-associating ionic/hydrogen bonding during bioprinting, its post-printing stability and tissue regeneration performance are not good enough in its applications. Gelatin derivatives has been employed as bioprinting materials by many companies worldwide, but this method requires photochemical cross-linking agents, which have still biocompatibility issues. Mixture of poly (ethylene glycol) and silk with/without stem cells were reported as a self-standing bioink in 3D bioprinting and injectable gel for its application to cartilage regeneration [33, 34].

In this study, we evaluated a hydrogel for its potential application as a HA-g-pHEA-gelatin bioink, consisting of three biocompatible biomaterials such as HA, HEA and gelatin. This hydrogel was obtained by graft polymerization of HEA to HA and then grafting of gelatin-methacrylate via radical polymerization mechanism as reported in our previous paper. While HA has been reported as an important natural polymer for its applications to tissue regenerations such as cartilage, bone and blood vessel, gelatin has been employed for potentially higher cell adhesion and proliferation as a key polymeric component in terpolymer for tissue engineering. HEA has been employed as medical device polymers such as poly (hydroxyethyl acrylate). To utilize these properties of 3 components, we evaluated its potential as a bioink by expecting biocompatibility, mechanical properties by HEA and gelatin. Physical and biological properties of this hydrogel fabricated with different concentrations of methacrylic anhydride (6 and 8%) have demonstrated excellent properties such as good swelling, rheology, gel morphology, cyto-compatibility, and delivery of small molecular drug such as DMOG, even though there were no significant effects of its concentrations on their properties (4, 6 and 8%). These reasons have been reported to be the effects of saturation of methacrylate graft to gelatin [35]. After verifying its physical and biological properties, HA-g-pHEA-Gelatin gel as bioink with bone cell loaded were bioprinted in lattice forms by using home-built multi-material (3D bio-) printing system. The experimental results demonstrated that the HA-g-pHEA-Gelatin hydrogel showed both stable rheology properties and excellent biocompatibility, and the gel showed printability in good shape.

## Conclusion

The 3D printing of HA-g-pHEA-Gelatin hydrogel was successful and the bone cells in bioinks were viable, when printed in lattice forms. The three component hydrogel was biocompatible and gel printing processing was excellent. This study demonstrated the HA-g-pHEA-Gelatin gel has a potential to be used as a bioink or its tissue engineering applications.
